# Solitary pancreatic metastasis of extremity myxoid liposarcoma: a case report and literature review

**DOI:** 10.1186/s12885-018-5059-1

**Published:** 2018-11-16

**Authors:** Dingding Wang, Jie Wu, Jian Yu, Hong Zhang, Honggang Liu

**Affiliations:** 1 Department of Pathology, Beijing Tongren Hospital, Capital Medical University, Beijing, 100730 China; 2 Department of Pathology, The Affiliated Hospital of Qingdao University, Qingdao, 266003 Shandong China; 3 Department of Oncology, Rizhao Central Hospital, Rizhao, 276800 Shandong China

**Keywords:** Myxoid liposarcoma, Metastasis, Pancreas, Pancreatectomy, Sarcoma

## Abstract

**Background:**

Myxoid liposarcoma has a distinct migration aptitude; however, pancreatic metastasis is rare.

**Case presentation:**

We report on the case of a 40-year-old female patient who suffered solitary pancreatic metastasis of myxoid liposarcoma and had a right thigh myxoid liposarcoma radical resection 5 years ago. The patient underwent a medial pancreatectomy and pancreaticojejunostomy for solitary pancreatic metastasis of myxoid liposarcoma. After 12 months of disease-free survival, the patient underwent an extended radical resection for the recurrence of the right thigh primary myxoid liposarcoma and received postoperative radiotherapy. Currently, the disease-free survival time after the last operation has been 22 months.

**Conclusions:**

We reviewed the relevant literature and suggest that radical surgery might result in a good prognosis for patients with solitary pancreatic metastasis of myxoid liposarcoma.

## Background

Liposarcoma (LPS) is a common mesenchymal tissue malignant tumor, which accounts for 12.8% of sarcoma and mainly occurs in the extremities and retroperitoneal [1]. Based on the World Health Organization classification of tumors [2], LPS are divided into well-differentiated, dedifferentiated, myxoid/round-cell, and pleomorphic LPS. Myxoid/round-cell liposarcoma (MLPS/RCLPS) is a subtype of soft tissue LPS (accounting for 20% of LPS), which occurs mainly in teenagers and in the thigh region [3, 4]. MLPS is a mesenchymal malignant tumor composed of uniform round to oval primitive non-lipogenic mesenchymal cells and some small signet-ring lipoblasts in a myxoid stroma with a characteristic branching vascular pattern [5]. RCLPS is a phenotype of MLPS, which is defined as having a greater than 5% round cell component in a MLPS. Based on a round cell component of greater or less than 5% of tumor volume, tumor biological behavior has been designated as either low or high grade, respectively, in previous studies [6].

MLPS is different from other subtypes as it shows an obvious migration tendency, especially for extrapulmonary metastasis [3, 7–10]. However, metastasis of the pancreas is unusual. Following the first report of MLPS by Carboni et al. [11], we present the second reported case of a patient with resected solitary pancreatic metastasis of extremity MLPS, who underwent a metastasectomy. We review the relevant literature and discuss feasible methods of treatment for this disease.

## Case presentation

The patient was a female, 40 years of age, who was admitted to hospital for a pancreatic body lesion. The pancreatic lesion was found by an abdominal ultrasound examination, and she was completely asymptomatic. The patient had a previous caesarean operation 6 years ago and had no previous trauma. Five years ago, she underwent right thigh LPS radical resection without any adjuvant treatment. The postoperative pathological examination revealed a well-differentiated MLPS, in which a lump of 8 cm in the upper abdomen could be felt combined with percussion pain, which moved moderately. The results from the laboratory examination were normal, including for tumor markers (CA19–9, CEA, CA125, CA50, and CA242), biochemical, and routine blood tests.

**Fig. 1:**
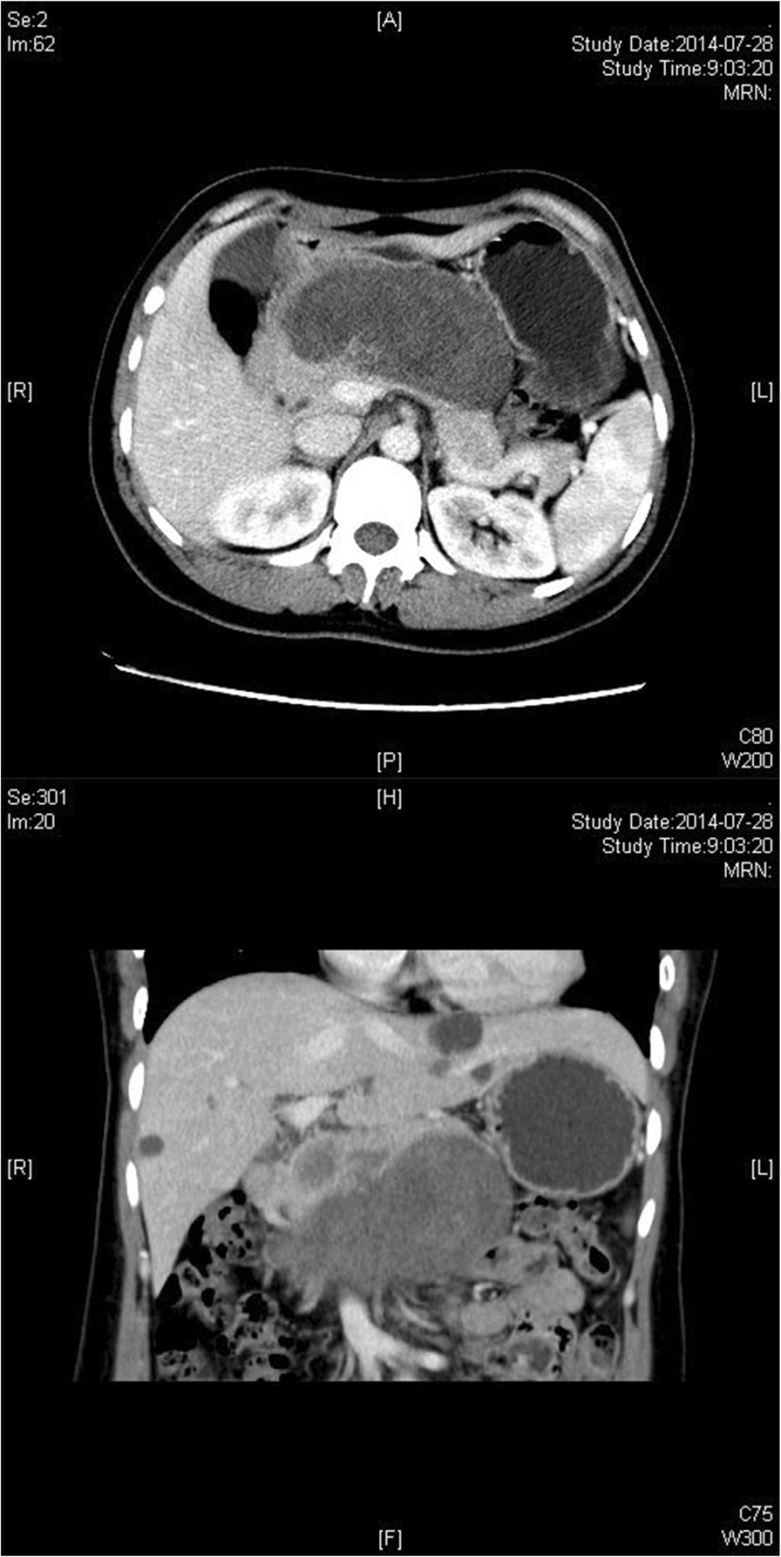
Abdominal dynamic enhanced CT showing a solid and cystic tumor in the body of the pancreas

**Fig. 2:**
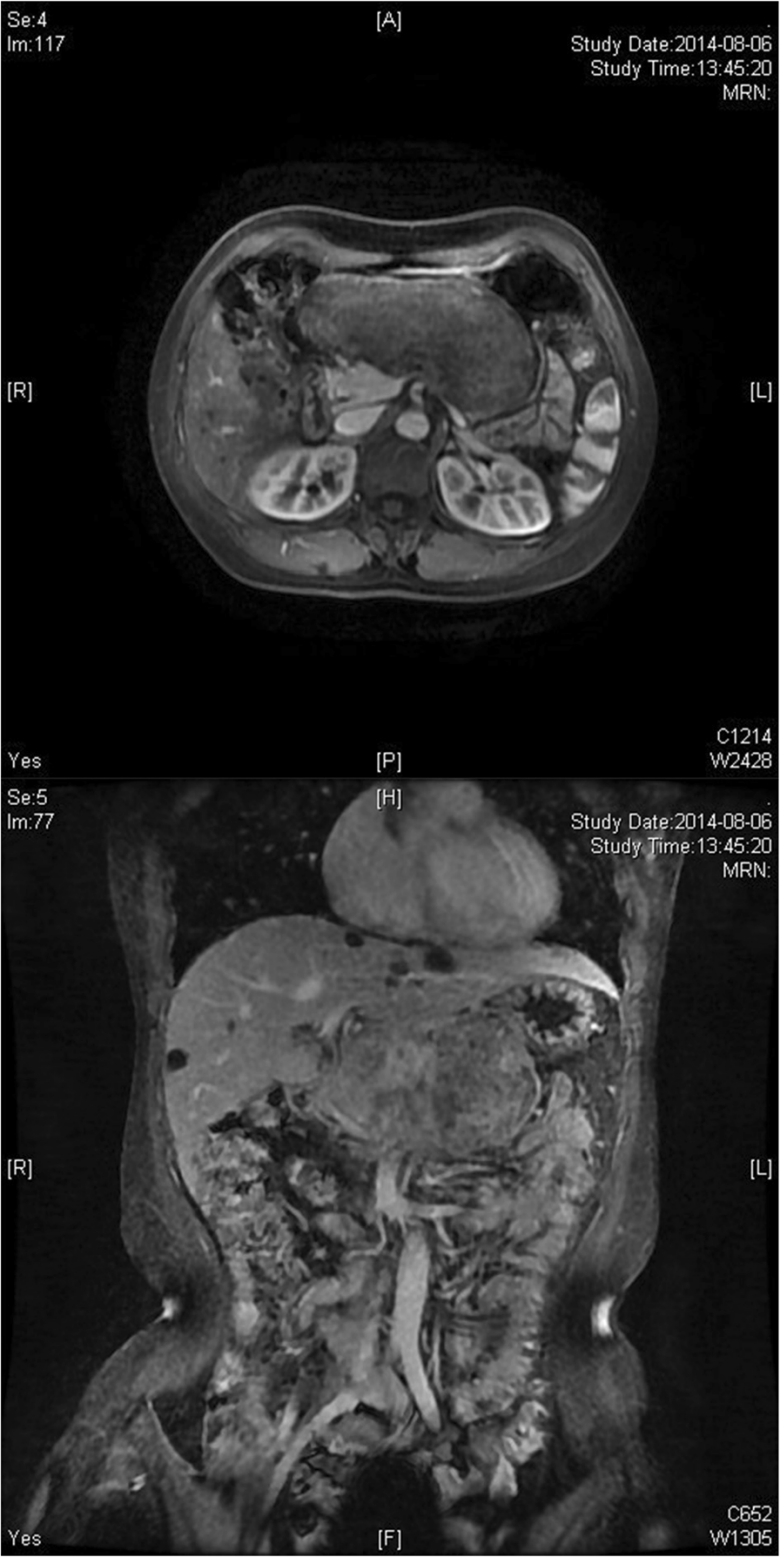
Abdominal dynamic enhanced MRI showing a long T1 signal shadow lesion in the body of the pancreas

An abdominal ultrasound (US) revealed a hypoechoic tumor in the upper abdomen (13.7 × 4.5 × 9.1 cm in size), which showed striped blood flow signals and could not be separated from pancreatic parenchyma. Abdominal dynamic enhanced computed tomography (CT) revealed a solid and cystic tumor in the body of the pancreas (10.4 × 4.9 cm in size) and the solid portion of the tumor was clearly enhanced (Fig. 1). Abdominal dynamic enhanced magnetic resonance imaging (MRI) revealed a long T1 signal lesion in the body of the pancreas, which showed edge enhancement during the balance and delay period, whereas the centre of the lesion did not (Fig. 2).

**Fig. 3:**
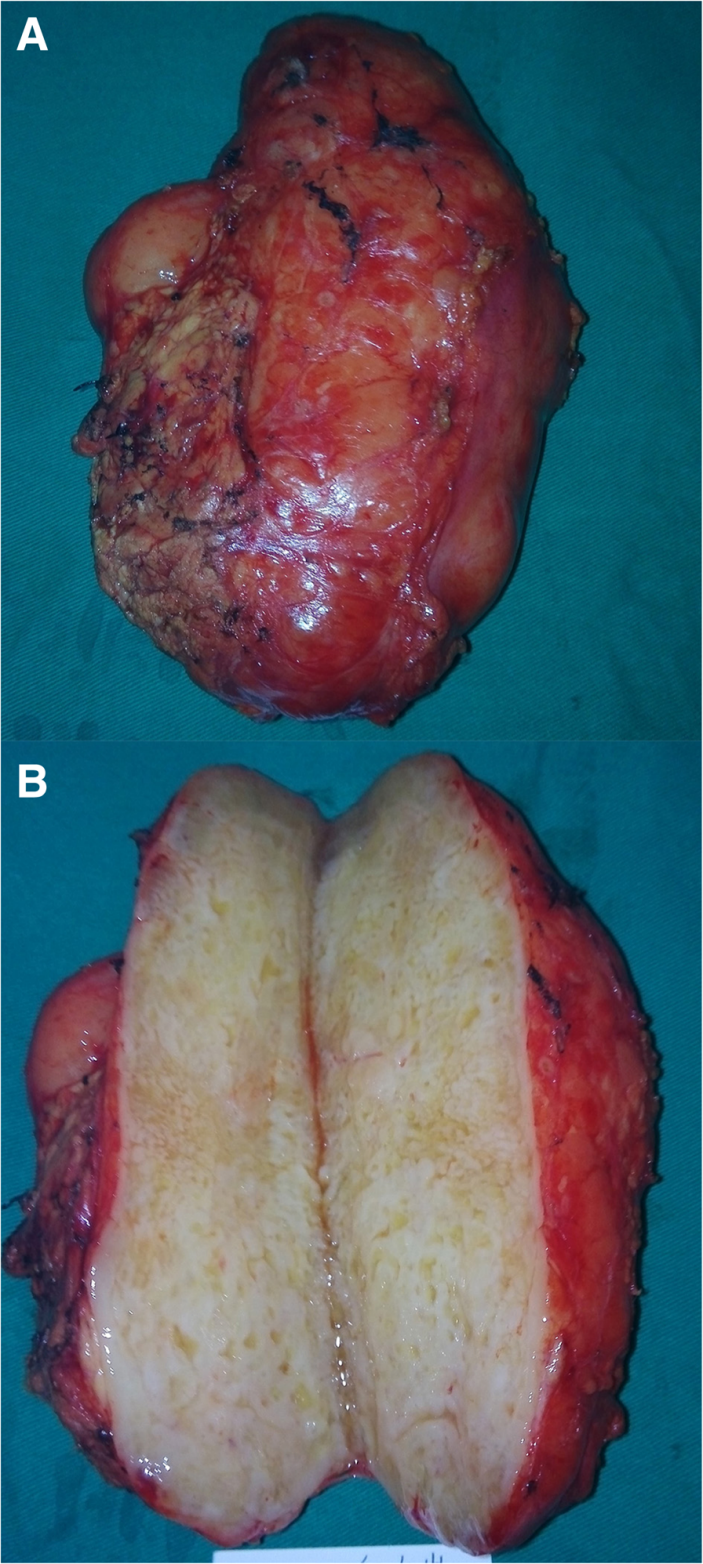
**a** The grey neoplasm approximately 14 × 9 × 7 cm in size. **b** The cut surface of the tumor was gray, friable, honeycomb-shaped, and muculent

**Fig. 4:**
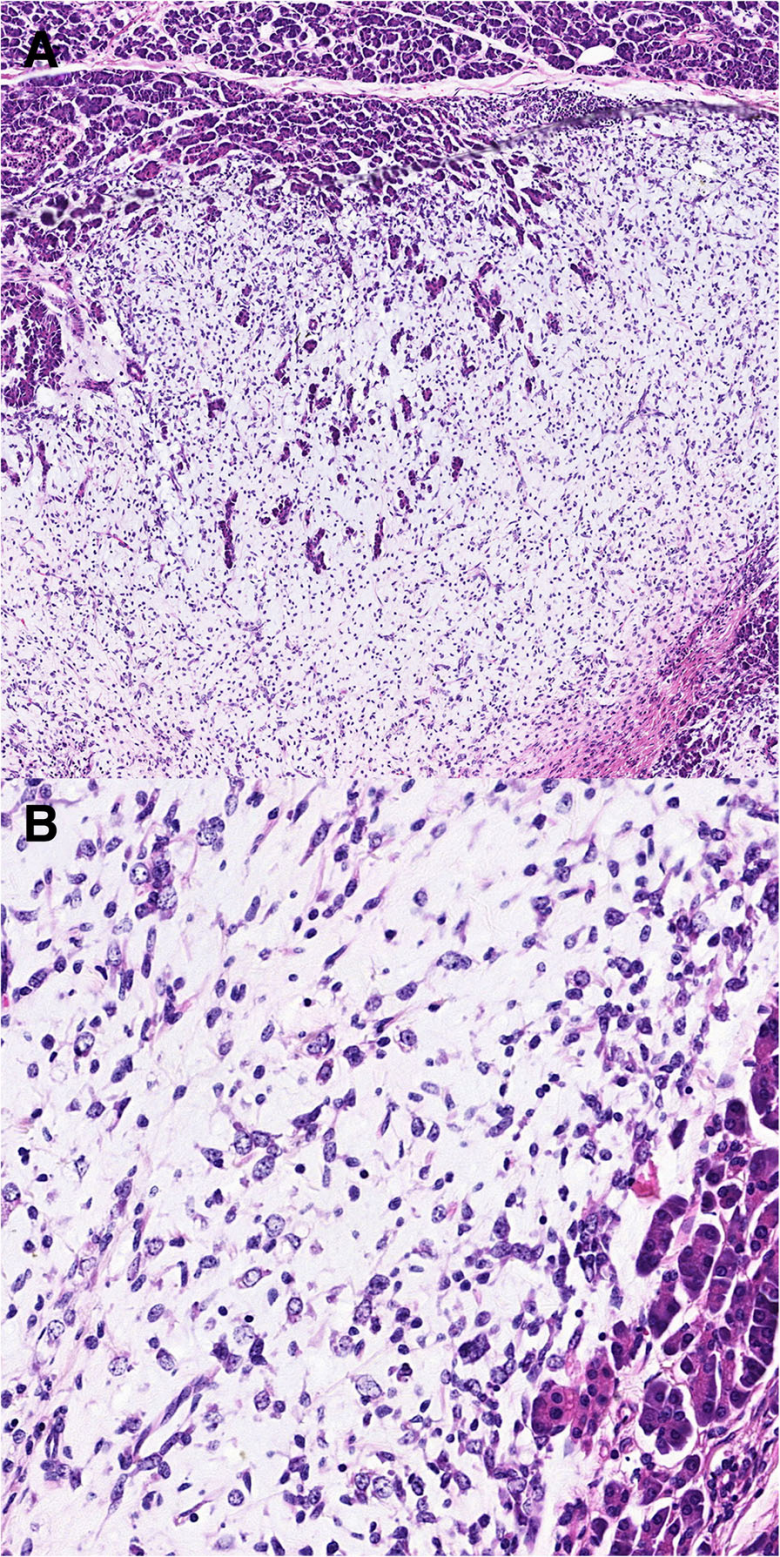
Pathological examination: **a** Low degree malignant spindle cell tumor combined with edema and mucoid degeneration; adipocyte and lipoblastoma were found; no round-cell components were found; and the tumor had infiltrated the surrounding pancreatic tissue (HE × 100). **b** The nucleus had a mild, strange type (HE × 400)

The preoperative imaging diagnosis of the lesion was pancreatic cystadenoma in the body of the pancreas, and an active pancreatectomy was performed. During the operation, we found a large tumor in the body of the pancreas, and performed a medial pancreatectomy and pancreaticojejunostomy. From the gross inspection, the grey neoplasm was approximately 14 × 9 × 7 cm in size (Fig. 3a), and there was no clear boundary between the tumor and pancreatic tissue. The cut surface of the tumor was gray, friable, honeycomb-shaped, and muculent (Fig. 3b). The pathological examination showed a low degree malignant spindle cell tumor combined with edema and mucoid degeneration, in which the nucleus had a mild, strange type; no round-cell components were found; the tumor had infiltrated the surrounding pancreatic tissue; and the surgical margin was negative (Fig. 4a). The final pathological diagnosis was MLPS (low degree malignant). The immunohistochemical results showed S-100(−), NSE(+), CD117(+), CD34(−), CD99(+), Dog-1(−), SMA(−), EMA(−), CK(−), Vim(+), HMB45(−), and the positive rate of Ki67 was 8% (Fig. 4b).

Pancreatic leakage occurred on the eighth day after surgery, although this complication had completely disappeared after 2 months due to effective drainage. The postoperative re-examination was negative from both the abdominal CT and US. However, after 12 months of disease-free survival time, the patient underwent a right thigh LPS extended radical resection for primary recurrence at the same site of primary tumor and received a postoperative radiotherapy of 60 Gy to her right thigh. The pathological examination revealed MLPS (low degree malignant). Currently, the disease-free survival time after the last operation has been 22 months.

## Discussion and conclusions

MLPS is the most common LPS in adolescents, and the majority of MLPSs occur in the extremities and rarely occur in the retroperitoneum [3, 4]. MLPS is likely to undergo both local recurrence and distant metastasis. In several previous studies, the distant metastatic rate of pure or no more than 5% round cell MLPS varied from 5 to 32% [7–9, 12, 13]. MLPS represents an uncommon tendency of extrapulmonary metastasis, which is different from other types of LPS. Previous studies have reported that the common extrapulmonary metastatic sites of MLPS (17–30%) include the retroperitoneum, subcutaneous soft tissue, bone, abdomen, and chest wall [3, 7–9]. Meanwhile, other studies have reported that MLPS represents a unique tendency to metastasize to other soft tissue sites, such as the retroperitoneum, thorax, and extremities, before spreading to the lungs [6]. Hoffman et al. [14] reported that the abdomen (49%) was a more common metastatic site for MLPS than the lungs (14%) and bones (23%). The low pulmonary metastatic potential of MLPS provides patients with a relatively long survival time [15]. This also allows abdominal metastatic tumors to increase in size. It is necessary to perform chest and abdominal CT during the postoperative re-examination to discover metastasis [7–9, 16]. Several studies reported that a metastasectomy could significantly improve prognosis in patients with metastasis, and a R0 resection can promote long-term survival [7, 8, 16, 17].

Malignant tumor pancreatic metastasis is rare, and previous studies have reported that only 2–5% of pancreatic malignant tumors are metastases [18]. Minni et al. [19] reported that 64.7% of pancreatic metastasis was solitary and mainly located in the head of the pancreas. Sarcoma combined with pancreatic metastasis is even rarer. Yoon at el. [20] reported that only 4% of patients had sarcoma pancreatic metastasis during an analysis of 53 pancreatic metastasis patients. MLPS combined with pancreatic metastasis is rare; to the best of our knowledge this is only the second time that MLPS combined with resected solitary pancreatic metastasis has been reported, following the report by Carboni et al. [11].

Owing to similar clinical symptoms, in the majority of cases it is difficult to identify a differential diagnosis between pancreatic metastasis and the primary tumor [19, 21]. In previous studies, 50% of patients were asymptomatic and were only discovered in re-examination by accident [22]. Thus, although pancreatic metastasis rates are low, attention should be given to patients who have a pancreatic tumor with a history of malignancy [16, 21]. Even though, post-surgery, the disease-free survival time is long, pancreatic metastasis still needs to be excluded especially for soft tissue sarcoma [11]. Long-term outcomes following pancreatic metastasectomies are greatly dependent on tumor biology [23]. Therefore, it is important to identify diagnosis for surgery, which may increase long-term survival. Nevertheless, even when the diagnosis is uncertain, the decision to proceed with a pancreatectomy is often made because the possibility of a pancreatic primary cannot be excluded [23].

The CT appearance of most pancreatic metastasis is of low density, and the enhancement CT appearance is edge enhancement in the arterial and venous phases [19]. The MRI appearance of pancreatic metastasis has long T1 and T2 signals, and the enhancement MRI appearance is also edge enhancement in the arterial and venous phases [17]. Owing to their high water content, MLPS are often pathognomonically present with long T1 and T2 signals in the MRI. Sheah et al. [10] reported that the MRI and enhancement MRI are the most sensitive methods for the evaluation of MLPS metastases. Besides, an FDG-PET scan is not recommended to detect metastases of MLPS as it has limited diagnostic value for MLPS metastases [3, 10]. Meanwhile, Seo et al. [24] reported that a whole-body MRI is a feasible examination for the detection of MLPS metastases. De Witt et al. [25] reported that 88% of pancreatic metastasis revealed a single hypoechoic lesion from an US, and the US can also discover 17% pancreatic metastasis, which were negative from the CT. However, the diagnosis of pancreatic metastasis should not only depend on diagnostic imaging. In our case, although we performed an US, enhancement CT, and MRI, we still made a misdiagnosis before the operation. In the case of Carboni et al. [11], a fine needle aspiration (FNA) was performed and a definitive preoperative diagnosis was identified. It has been reported that the accuracy of endoscopic ultrasound (EUS) FNA is approximately 89% in pancreatic metastases [26]. Thus, CT, US, or EUS guided FNA can efficiently make a definitive diagnosis.

Standardized pancreatic resections including partial pancreaticoduodenectomy, distal pancreatectomy, and total pancreatectomy are recommended for the management of isolated pancreatic metastases [23]. In the past, it was believed that pancreatectomy had high mortality and morbidity rates; however, recent clinical analyses of large sample sets have revealed that a pancreatectomy has low mortality rates and acceptable morbidity rates, and the operation for pancreas metastases is safe [17, 21, 27]. Xiao et al. [28] reported that medial pancreatectomy maintains pancreatic endocrine and exocrine function better than other standardized pancreatic resections, which is suitable for benign or low grade malignant lesions. In previous literatures, the perioperative mortality rate has been reported as being 1–3% [23]. Although the morbidity rate is relatively high, most complications could be completely cured by non-operative management [27]. Sperti et al. [21] reported that intraoperative US could guide the operation area and measure the distance between the metastasis tumor and pancreatic duct. Zerbi et al. [17] reported that a R0 resection could obtain radical therapy for pancreatic metastasis and other invaded tissue, except the pancreas, if technology was allowed to resect. Several studies have reported that most pancreatic metastases after a radical resection obtained a favorable prognosis, especially for patients with long disease-free survival times after primary surgery [19, 21]. Therefore, the effectiveness of a pancreatectomy is highly dependent on the tumor biology of the primary cancer [23].

A wide resection with a negative margin of at least 10 mm of adjacent tissue is the standard treatment for primary MLPS, and neoadjuvant or adjuvant treatment using radiotherapy and/or isolated limb perfusion may allow smaller margins [4, 29, 30]. Prognostic factors of MLPS have been described in several previous studies. The percentage of round cell component (*≥* 5%), the presence of tumor necrosis and old age (> 45 years) were significantly associated with metastases and a poor prognosis [2, 6, 31–33]. Unfortunately, LPS combined with pancreatic metastasis is rare and there is no related prognostic research. Reddy et al. [34] reported that, in a prognostic analysis of 10 patients with pancreatic metastasis of sarcoma who underwent pancreatectomy, the median survival time was 40 months and the 5-year survival rate was 14%. Robert et al. [35] also reported a prognostic analysis of 17 patients with pancreatic metastasis of leiomyosarcoma. In the 17 patients, 7 underwent an operation and 5 experienced long-term survival, with a median disease-free survival time of 23 months. Sperti et al. [27] reported that a pancreatectomy was suitable for individual patients with pancreatic metastasis of sarcoma. No prognostic research has been previously undertaken; however, previous studies have shown that MLPS has a good prognosis and a metastasectomy can improve the prognosis in patients with metastasis of other organs [7, 8, 14, 16, 17]. Especially in patients with low-grade, slow-growing LPS, a metastasectomy might allow disease control over many years [4]. Both in the present study and in Carboni et al.’s [11], the pathology pattern belonged to pure MLPS and pancreatic metastases were solitary, which were designated as low grade. The months between extrapulmonary recurrences were 60 and 72 months, respectively, which revealed slow disease progression. In the study of Hoffman et al. [14], the indications of an MLPS metastasectomy included low tumor grade, age < 50 years, tumor size < 1.5 cm, and slow disease progression (> 12 months between pulmonary recurrences or > 24 months between extrapulmonary recurrences). The appropriate prognosis of MLPS allows abdominal metastatic tumors to increase in size [15]. Thus, we believe that patients with resected MLPS pancreatic metastasis, especially with low tumor grade and slow disease progression, undergoing a pancreatectomy might lead to long-term survival on the basis of current information.

The effect of radiotherapy has been demonstrated in the local lesions of MLPS [12] and the rates of radiotherapy receipt have also increased [13]. Although short-term wound complications are common, the late effects of radiotherapy are substantially less frequent [36]. Prestwich et al. [37] suggested that radical radiotherapy should be used in the multimodality treatment of MLPS metastases.

Chemotherapy in selected patients with MLPS has obtained positive response rates and improved local and distant control rates, although it has been shown not to prolong survival [14]. MLPS is considered most responsive to cyclophosphamide, with remission rates of up to 50% [38]. Of note, MLPS is sensitive to trabectedin, with a response rate of 51% even in pretreated patients, which may justify the use of trabectedin, even in first-line therapy when a clinical situation requires tumor shrinkage [39]. Due to the insidious symptom, some pancreatic metastases were diagnosed to be unresectable [27]. Thus, in patients with fast-growing, high-grade sarcomas and unresected solitary or multiple metastases, systemic chemotherapy remains the mainstay of treatment [4].

The reason for the metastatic tendency of MLPS is not clear. Ogose et al. [40] reported that the unusual extrapulmonary metastatic tendency of MLPS might be correlated with an abundance of fat cells in the metastatic sites, such as subcutaneous tissue, retroperitoneum, bone marrow, and epidural space. Meanwhile, Hoffman et al. [14] reported that there were several highly expressed molecules in MLPS, such as adipophilin, peroxisome proliferator-activated receptor-γ (PPAR-γ), chemokine (C-X-C motif) receptor 4 (CXCR4), AXL receptor tyrosine kinase, and platelet-derived growth factor receptor-β (PDGFR-β), which are associated with adipogenesis, migration, invasion, angiogenesis, and metastasis.

In conclusion, for patients with solitary pancreatic metastasis of soft tissue sarcoma, especially with low tumor grade and slow disease progression, radical surgery might offer a good prognosis.

## Abbreviations

CT: Computed tomography
CXCR4: Chemokine (C-X-C motif) receptor 4
EUS: Endoscopic ultrasound
FDG-PET: Fluorodeoxyglucose positron emission tomography
FNA: Fine needle aspiration
LPS: Liposarcoma
MLPS: Myxoid liposarcoma
MRI: Magnetic resonance imaging
PDGFR-β: Platelet-derived growth factor receptor-β
PPAR-γ: Peroxisome proliferator-activated receptor-γ
RCLPS: Round cell LPS
US: Abdominal ultrasound
